# Efficacy of prothrombin complex concentrates for the emergency reversal of dabigatran-induced anticoagulation

**DOI:** 10.1186/s13054-016-1275-8

**Published:** 2016-04-28

**Authors:** Oliver Grottke, James Aisenberg, Richard Bernstein, Patrick Goldstein, Menno V. Huisman, Dara G. Jamieson, Jerrold H. Levy, Charles V. Pollack, Alex C. Spyropoulos, Thorsten Steiner, Gregory J. del Zoppo, John Eikelboom

**Affiliations:** Department of Anesthesiology, RWTH Aachen University Hospital, Pauwelsstrasse 30, 52074 Aachen, Germany; The Icahn School of Medicine at Mount Sinai, New York, NY USA; Northwestern University Feinberg School of Medicine, Chicago, IL USA; Emergency Department and SAMU, Lille University Hospital, Lille, France; Department of Thrombosis and Hemostasis, Leiden University Medical Center, Leiden, The Netherlands; Department of Neurology, Weill Cornell Medical College, New York, NY USA; Department of Anesthesiology, Duke University School of Medicine, Durham, NC USA; Department of Emergency Medicine, Thomas Jefferson University, Philadelphia, PA USA; Hofstra North Shore/LIJ School of Medicine, Lenox Hill Hospital, New York, NY USA; University of Heidelberg, Heidelberg, Germany; Departments of Medicine and Neurology, University of Washington, Seattle, WA USA; McMaster University, Hamilton, Ontario Canada

**Keywords:** Anticoagulation, Activated prothrombin complex concentrate, Bleeding, Dabigatran, Prothrombin complex concentrate, Trauma

## Abstract

Dabigatran is effective in decreasing the risk of ischaemic stroke in patients with atrial fibrillation. However, like all anticoagulants, it is associated with a risk of bleeding. In cases of trauma or emergency surgery, emergency reversal of dabigatran-induced anticoagulation may be required. A specific reversal agent for dabigatran, idarucizumab, has been approved by the US Food and Drug Administration. Alternative reversal agents are available, such as prothrombin complex concentrates (PCCs) and activated PCCs (aPCCs). In this review we evaluate the role of PCCs and aPCCs in the reversal of dabigatran anticoagulation and consider which tests are appropriate for monitoring coagulation in this setting. Pre-clinical studies, small clinical studies and case reports indicate that PCCs and aPCCs may be able to reverse dabigatran-induced anticoagulation in a dose-dependent manner. However, dosing based on coagulation parameters can be difficult because available assays may not provide adequate sensitivity and specificity for measuring anticoagulation induced by dabigatran or the countering effects of PCCs/aPCCs. In addition, PCCs or aPCCs can potentially provoke thromboembolic complications. Despite these limitations and the fact that PCCs and aPCCs are not yet licensed for dabigatran reversal, their use appears to be warranted in patients with life-threatening haemorrhage if idarucizumab is not available.

## Background

For more than 60 years, the only oral anticoagulants available for the prevention of ischaemic stroke in patients with non-valvular atrial fibrillation (AF) have been the vitamin K antagonists (VKAs), such as warfarin, phenprocoumon and acenocoumarol [1]. However, the need for coagulation monitoring and dose adjustments, as well as concerns about drug–drug or diet–drug interactions and the risk of bleeding, have restricted the use of VKAs for ischaemic stroke prevention in patients with AF [2, 3]. Several direct oral anticoagulants (DOACs) have been approved for this indication: the oral direct thrombin inhibitor dabigatran, and oral direct factor Xa inhibitors (e.g. rivaroxaban, apixaban or edoxaban) [4, 5]. Compared with VKAs, DOACs produce a more predictable anticoagulant effect and can be given in fixed doses without routine coagulation monitoring. The results of randomised controlled trials (RCTs) and observational studies indicate that DOACs are at least as effective as VKAs for stroke prevention in patients with AF, with reduced rates of intracranial bleeding and, at certain doses, a reduction in life-threatening bleeding [6–10].

Dabigatran, the active moiety of dabigatran etexilate, has a rapid onset of action, and the plasma concentration peaks within 0.5–2.0 hours of administration [11]. This anticoagulant has a half-life of 7–17 hours [12] and is eliminated predominantly via renal excretion (80 %) [13]. Dabigatran is licensed in many countries for several indications, including: primary prevention of venous thromboembolism (VTE) in patients who have undergone elective total hip or knee arthroplasty (150 mg or 220 mg once daily); prevention of ischaemic stroke and systemic embolism in adult patients with non-valvular AF (110 or 150 mg twice daily, except in the USA where 75 and 150 mg twice-daily doses are approved); and treatment of acute deep vein thrombosis (DVT) and pulmonary embolism (110 and 150 mg twice daily, except in the USA where only the 150 mg twice-daily dose is approved) [14, 15].

Most episodes of bleeding in patients treated with dabigatran can be managed with supportive measures and by temporarily withholding the drug. However, additional strategies may be needed in patients with life-threatening bleeding and those who require urgent or emergency surgery or other invasive procedures for which haemostasis is necessary. Supportive care is also not sufficient in patients with intracranial bleeding, where outcome may be directly associated with the time needed for coagulation reversal [16].

A specific reversal agent for dabigatran, idarucizumab, has been approved by the US Food and Drug Administration. Animal models and phase I–III clinical data show that idarucizumab achieves predictable, complete and sustained reversal of dabigatran, with the potential for significant reductions in blood loss [17–19]. In an interim analysis of the phase III RE-VERSE AD study [19], involving 90 dabigatran-treated patients with serious bleeding or in need of an urgent surgical procedure, idarucizumab completely reversed the effects of dabigatran within minutes. Idarucizumab appears to be well tolerated, and it has no direct effects on procoagulant or anticoagulant activity [17, 19]. However, idarucizumab is yet to be approved in many countries and, pending its widespread availability, multiple therapeutic options have been suggested for emergency reversal of dabigatran’s anticoagulant effects. These options include prothrombin complex concentrates (PCCs) and activated PCCs (aPCCs), as well as recombinant activated factor VII (rFVIIa) [20–24].

In this article we review pre-clinical and clinical evidence for the use of PCCs and aPCCs to restore haemostasis in dabigatran-treated patients with either haemorrhage or the need for an urgent surgical procedure, and review the role of laboratory coagulation assessments in this setting [20–24].

## Mechanism of action of dabigatran

**Fig. 1:**
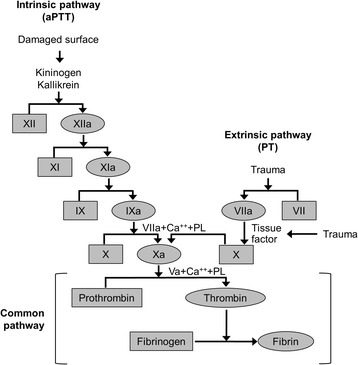
The coagulation cascade [27]. The common pathway shows the dependence of coagulation on the action of thrombin. Permission for reproducing this figure has been requested from John Wiley and Sons, Inc. *aPTT* activated partial thromboplastin time, *PL* phospholipase, *PT* prothrombin time

Dabigatran is a small molecule that binds competitively and selectively to the catalytic site of thrombin [25, 26]. Since thrombin facilitates coagulation by converting fibrinogen into a fibrin network, dabigatran has the effect of blocking the terminal coagulation cascade (Fig. 1) [27]. Additional effects of thrombin prevented by dabigatran include platelet activation, amplification of coagulation activation (positive feedback mechanism) and inhibition of fibrinolysis [25].

## Pre-surgical management of the dabigatran-anticoagulated patient

Because dabigatran has a short half-life (in patients with normal renal function) and its effects on thrombin are reversible, the general recommendation for dabigatran-treated patients scheduled to receive elective surgery is to discontinue therapy temporarily and wait for the remaining drug to be eliminated naturally. The timing of discontinuation before elective surgery is based on a patient’s renal function and risk of bleeding, and the type of surgery planned. In patients with normal renal function, dabigatran should be stopped at least 24 hours before surgery with a standard risk of bleeding; a 48-hour interim is appropriate if the risk of bleeding is high [15]. These time periods increase to 2–3 days and 4 days, respectively, in patients with reduced renal function (creatinine clearance ≥30–50 ml/min) [28].

## General bleeding management of the dabigatran-anticoagulated patient

Expert and professional society guidelines are available regarding emergency bleeding management in patients receiving dabigatran treatment [28–32]. There are variations in the specifics but the principles are consistent across different guidelines. The general approach to managing non-emergency bleeding complications is similar to that with pre-surgical management, i.e. discontinue dabigatran therapy temporarily and wait for the elimination of dabigatran. In this review, we will focus on major or life-threatening bleeding (including intracranial bleeding) and emergency surgery, where a need for more rapid reversal of the effects of dabigatran necessitates a different strategy. Exogenous coagulation factor repletion with PCCs or aPCCs has been suggested as a potential treatment option in these settings [33].

The initial steps of bleeding management algorithms typically consist of local/surgical haemostasis where appropriate, qualitatively assessing anticoagulant activity (e.g. by activated partial thromboplastin time (aPTT)), and general measures such as volume replacement and blood product transfusion. Medical history including anticoagulant intake is integral to the early phase of treatment. In many cases, however, anticoagulation may not be contributing meaningfully to the bleeding and so reversal of dabigatran is not usually a first-line priority. In the RE-VERSE AD study [19], 22 out of 90 enrolled patients did not have prolonged diluted thrombin times (dTTs) due to the natural clearance of dabigatran. Acquired coagulopathy can develop secondary to blood loss, loss and consumption of coagulation factors and haemodilution owing to excessive fluid replacement. This is a major risk factor for progression from initial bleeding to severe haemorrhage. There is some evidence suggesting that ‘restrictive’ or goal-directed as opposed to ‘liberal’ fluid resuscitation strategies may reduce morbidity and lengths of hospital stay [34, 35]. However, other investigations have cast doubt on the benefits of a restrictive approach [36] and, importantly, hypovolaemia can cause acidosis, thereby exacerbating coagulopathy. In the presence of substantial tissue injury and haemorrhagic shock, activation of protein C and subsequent hyperfibrinolysis may also aggravate coagulopathy [37]. Early intervention with haemostatic therapy (e.g. fibrinogen concentrate, cryoprecipitate, fresh frozen plasma, platelets) may be critical for preventing complex coagulopathies and progression to severe, life-threatening haemorrhage—especially in patients who have bled so that they have acquired coagulopathy in addition to anticoagulant therapy. Multimodal therapy should therefore be administered as early as possible in life-threatening bleeding under dabigatran anticoagulation [38].

## Laboratory evaluation of dabigatran concentration

Although patients taking dabigatran do not need to undergo routine coagulation monitoring, rapid assessment of whether or not the patient is actively anticoagulated is important in an emergency situation. This information can help determine the contribution of anticoagulation to the bleeding, the need for a reversal/repletion strategy and whether an invasive procedure should be delayed [39, 40].

A variety of tests have been explored for the detection or quantification of plasma dabigatran activity, but limitations are common. For example, the prothrombin time (PT) and the international normalised ratio (INR) exhibit low sensitivity to anticoagulation with dabigatran, with therapeutic doses having minimal effect; these tests are therefore not recommended [31]. Thrombin-generation assays and viscoelastic testing parameters have also been considered, but thrombin-generation assays are mostly restricted to research settings and viscoelastic testing has not been validated for the monitoring of dabigatran in a clinical setting [40]. The aim of testing for dabigatran activity is often not to provide precise quantification of dabigatran, but simply to detect the presence or absence of drug in plasma. This is particularly important in patients with acute ischaemic stroke, for whom thrombolytic therapy or procedures requiring high-dose antiplatelet agents are being considered. Candidate tests are now described in more detail and are summarised in Table 1.

**Table 1:** Clinical and research tests available for the assessment of plasma dabigatran concentration

Test	Sensitivity to plasma dabigatran concentration	Availability	Clinical recommendation
Thrombin time	Linear dose response; oversensitive	Widely available	Recommended for detection of presence or absence of dabigatran activity
Activated partial thromboplastin time (aPTT)	Non-linear dose response	Widely available	Recommended for semi-quantitative estimation of dabigatran activity; normal aPTT does not always exclude presence of dabigatran
Direct thrombin inhibition assays	Linear dose response; sensitive	Usually available in specialised centres	Recommended for measurement of plasma concentration; ECT usually a local assay because commercial kits not available; not validated for dabigatran
Diluted thrombin time			
Ecarin clotting time (ECT)			
Thromboelastometry (ROTEM^®^)/thrombelastography (TEG^®^) assays EXTEM/Rapid TEG	Sensitive	Limited availability	Potential measurement of effectiveness of treatment for dabigatran-induced anticoagulation; not validated for dabigatran
Thrombin-generation assays	Sensitive (lag time)	Limited availability	Potential measurement of effectiveness of treatment for dabigatran-induced anticoagulation; not validated for dabigatran
Prothrombin time or international normalised ratio	Low sensitivity	Widely available	Not recommended in this setting

The aPTT exhibits a non-linear dose response with increasing concentrations of dabigatran, plateauing at higher concentrations [31]. In addition, in some patients taking dabigatran, normal-range aPTT values have been reported when dabigatran is at trough levels [41]. This test is therefore not suitable for precise quantification, particularly at high or low dabigatran concentrations, but can provide an approximate indication of dabigatran levels [42]. The aPTT is readily available and therefore is commonly used to evaluate dabigatran activity or possible drug ingestion; normal aPTT results reduce the likelihood of therapeutic anticoagulation.

The thrombin time (TT) exhibits a very steep, linear dose response with increasing concentrations of dabigatran. This test can be considered excessively sensitive for the detection of dabigatran activity because samples may not clot at dabigatran concentrations above 100 ng/ml, a level within the expected clinical range [41, 43]. Therefore, the use of this assay for quantifying therapeutic concentrations of dabigatran is limited. However, the TT is a very useful assay to determine the presence of low levels of dabigatran, with TT values within the normal range suggesting the absence of dabigatran.

The TT and aPTT assays are useful tests in the clinical setting and can be used in combination, with the TT detecting the presence or absence of drug and the aPTT providing an approximate indication of the plasma dabigatran concentration. These assays are readily accessible and can provide information rapidly.

Tests that are used principally for research have also been considered for assessing dabigatran anticoagulation. For example, dedicated direct thrombin inhibition assays have a linear relationship over a wide range of plasma dabigatran concentrations [44]. The dTT is one type of direct thrombin inhibition assay, in which plasma is diluted in buffer and then supplemented with normal human plasma; clotting is then initiated with thrombin. This methodology compensates for any coagulation factor deficiencies and only the effect of the direct thrombin inhibition is measured [45]. Ecarin tests, representing another type of direct thrombin inhibition assay, are either clotting based (ecarin clotting time (ECT)) or chromogenic (ecarin chromogenic assay (ECA)) [31, 45]. Ecarin is snake venom that directly activates prothrombin to initiate clotting, thereby bypassing upstream coagulation and enabling direct measurement of the influence of the direct thrombin inhibition. Some ECA tests are supplemented with prothrombin to allow targeted direct thrombin inhibition quantification independent of any coagulopathies in the patient sample. In a recent study, the dTT, ECT and ECA all showed linear correlations with dabigatran concentrations over a broad range [41]. Although these tests are widely available, they are not used in all hospitals [46].

The thrombin-generation parameter ‘lag time’ and, to some extent, the endogenous thrombin potential (ETP) have been shown to correlate with plasma dabigatran concentrations [47]. Recent studies have also shown that therapeutic dabigatran doses have a significant effect on the lag time, ETP and peak height, when measured by the calibrated automated thrombogram (CAT) [48]. However, thrombin-generation tests are time-consuming and their availability is generally limited to research laboratories and centres specialising in haemostasis. Thrombin-generation parameters therefore cannot be recommended for routine evaluation of plasma dabigatran concentration.

Viscoelastic tests, including thromboelastometry (ROTEM^®^) or thrombelastography (TEG^®^), can have faster turnaround times than standard laboratory coagulation tests because they are whole-blood based and are often performed in surgical or emergency rooms. This can be beneficial in emergency situations. The ROTEM^®^ EXTEM and Rapid TEG (r-TEG) assays measure tissue-factor-initiated extrinsic coagulation and pre-clinical data have shown that they are sensitive to dabigatran [49]. There is anecdotal evidence that dabigatran prolongs the activated clotting time (ACT) in r-TEG, when all other standard coagulation tests were within the normal range [50]. However, neither EXTEM nor r-TEG has been calibrated for measuring dabigatran levels; in many centres these assays are not used routinely and there is currently no clinical evidence supporting the use of these tests for this purpose.

## Prothrombin complex concentrates (activated and non-activated)

If the patient continues to sustain major blood loss after initial intervention with haemostatic therapy, or in the setting of intracranial haemorrhage, treatment algorithms recommend further interventions to counteract the anticoagulant effects of dabigatran (Table 2) [28–30, 32]. These interventions include PCCs and aPCCs.

**Table 2:** Recommendations and algorithms for the management of bleeding patients with dabigatran-induced anticoagulation

Reference	Mild bleeding	Moderate-to-severe bleeding	Life-threatening bleeding or intracranial haemorrhage
Weitz et al., 2012 [32]	Discontinue treatment until bleeding resolves	Sequential treatment:	aPCC (50 IU/kg)
		(1) PCC (40 IU/kg)	If unavailable, give PCC (40 IU/kg) or rFVIIa (90 μg/kg)
		(2) aPCC (50 IU/kg)	
		(3) rFVIIa (90 μg/kg)	
		(4) Haemodialysis for 6–8 h or charcoal filtration	
Faraoni et al., 2015 [29]	No recommendation given	No recommendation given	(1) Monitor blood loss and perform coagulation assays
			(2) Standard resuscitation with fluid therapy, tranexamic acid (1 g), RBCs and massive transfusion protocol^a^
			(3) Four-factor PCC (25–50 IU/kg), aPCC (FEIBA; 30–50 IU/kg)
EHRA guidelines [30]	Maintain diuresis	Same recommendation as for mild bleeding	PCC 50 U/kg (additional 25 U/kg if clinically needed)aPCC 50 U/kg (maximum 200 U/kg/day)rFVIIa (90 μg/kg)Idarucizumab 5 g intravenously
	Local haemostatic measures		
	Fluid replacement		
	RBC substitution if necessary		
	Platelet substitution if necessary		
	FFP as plasma expander (not as reversal agent)		
	Consider tranexamic acid or desmopressin		
	Consider dialysis		
ESA guidelines [28]	No recommendation given	No recommendation given	PCC, aPCC or rFVIIa may be used as non-specific antagonists

^a^Transfusion of FFP/platelets/RBCs

*aPCC* activated prothrombin complex concentrate, *EHRA* European Heart Rhythm Association, *ESA* European Society of Anaesthesiology, *FFP* fresh frozen plasma, *PCC* prothrombin complex concentrate, *RBC* red blood cell, *rFVIIa*, recombinant activated factor VII, *FEIBA*, factor eight inhibitor bypassing activity

A number of PCCs are commercially available. Detailed analysis of constituent differences between these products has been published previously [51]. All PCCs contain the vitamin K-dependent factors II, IX and X, and are standardised according to their factor IX content. In addition, they contain differing amounts of factor VII; products with low or high quantities of factor VII are known as three-factor or four-factor PCCs, respectively. Some PCCs also contain anticoagulation proteins such as protein C, protein S, protein Z, antithrombin and heparin. Furthermore, aPCCs are available which contain non-activated factors II, IX and X, and activated factor VII.

VKAs (e.g. warfarin) produce their anticoagulant effects by inhibiting the synthesis of vitamin K-dependent coagulation factors II, VII, IX and X. In patients with life-threatening bleeding, rapid replacement of these coagulation factors is required and therefore PCCs are a reasonable option. Both three-factor and four-factor PCCs have been investigated for the reversal of VKAs; four-factor PCCs are more commonly used, because three-factor PCCs do not provide adequate reductions in INR owing to the low levels of factor VII [52]. The first four-factor PCC was approved in the United States in 2013, specifically for this purpose [53]. Nevertheless, in VKA-associated bleeding, data for PCCs are based principally on laboratory rather than clinical endpoints, meaning that the evidence may be considered not to be at the highest level.

For the treatment of dabigatran-induced anticoagulation, neither PCCs nor aPCCs act as specific reversal agents for dabigatran or any other DOAC. Instead, they raise levels of the vitamin K-dependent coagulation factors, notably prothrombin, and thrombin generation is consequently increased. In the case of dabigatran, the plasma concentration of thrombin is increased to a stoichiometric excess vs the drug, and therefore levels of free (unbound) dabigatran and the antithrombotic effect of the drug are minimised. Data from pre-clinical and clinical studies of PCCs and aPCCs for reversal of dabigatran (see later sections) are consistent with this mechanism of action.

Although a number of suitable tests for the monitoring of plasma dabigatran concentration have been identified, it does not necessarily follow that these are the most appropriate tests for monitoring the reversal of dabigatran-induced anticoagulation by PCCs or aPCCs. For example, the aPTT may be useful in providing an approximate indication of dabigatran levels, but appears to be insensitive to the reversal effects of PCCs and aPCCs [49]. There is some evidence to suggest that the EXTEM assay parameters clotting time (CT) and clot formation time (CFT) are sensitive to the effects of PCCs and aPCCs on dabigatran anticoagulation; therefore, these parameters may potentially provide a means of monitoring dabigatran reversal, although clinical studies are warranted [49].

## Efficacy of PCCs and aPCCs for treating dabigatran-induced anticoagulation

The efficacy of PCCs/aPCCs to treat dabigatran-induced anticoagulation has been investigated in several pre-clinical studies, clinical studies and case reports; these are summarised in this section and in Tables 3, 4 and 5.

**Table 3:** Pre-clinical studies investigating the use of PCCs and aPCCs to reverse dabigatran-induced anticoagulation

Reference	Study design	Dose		Main results	Conclusion
		Dabigatran (mg/kg)	PCC (IU/kg)		
Zhou et al., 2011 [54]	Murine intracerebral haemorrhage model	9.0	100	Haematoma volume:	PCC effectively prevented haematoma growth and significantly reduced 24-h mortality
				Post-dabigatran: 17.0 ± 4.1 mm^3^	
				Post-PCC: 11.7 ± 3.0 mm^3^	
				Mortality:	
				Control animals: 30 %	
				PCC-treated mice: 4 %	
Pragst et al., 2012 [55]	Leporine standardised kidney injury model	0.4	20, 35 or 50	Blood loss:	PCC resulted in a dose-dependent reduction in blood loss and acceleration in haemostasis. At the highest dose, blood loss was normalised in all animals. All doses of PCC successfully treated dabigatran-induced anticoagulation at plasma concentrations similar to those seen in patients receiving dabigatran
				Control: 1.0–7.2 ml	
				Post-dabigatran: mean 29 ml	
				Post-PCC: decreased by 5.44 ml per 10 IU/kg PCC	
				No change in aPTT	
				PT shortened by 0.335 s per 10 IU/kg PCC	
Herzog et al., 2014 [56]	Leporine arterial venous shunt model	0, 0.075, 0.2, 0.45	0, 5 or 300	Bleeding time:	Dabigatran-induced bleeding was effectively reversed by PCC. The thromboembolic risk associated with PCC administration appeared to be reduced due to the persistence of dabigatran in the plasma
				Increasing PCC doses shortened time to haemostasis for rabbits treated with 0.2 mg/kg dabigatran	
				No dose of PCC could reverse the effects of 0.45 mg/kg dabigatran on time to haemostasis	
				Thrombosis:	
				The frequency of pulmonary thrombi decreased progressively with increasing concomitant dabigatran dose	
Grottke et al., 2014 [49]	Porcine liver trauma model	30 (daily oral dose) then intravenous infusion to reach supratherapeutic plasma concentration	PCC:	PCC:	Both PCC and aPCC diminished the effects of dabigatran, restoring ROTEM^®^ parameters and PT to 80–90 % of baseline
			30 or 60	No effect on aPTT	
			aPCC:	aPCC:	
			30 or 60	No effect on aPTT	
Honickel et al., 2015 [57]	Porcine polytrauma model	30 (daily oral dose) then intravenous infusion to reach supratherapeutic plasma concentration	aPCC:	50 IU/kg aPCC associated with significant reduction in blood loss vs placebo group and those treated with 25 IU/kg	aPCC (50 IU/kg) is effective in reducing blood loss in anticoagulated pigs
			25 or 50		
					Lower-dose aPCC (25 IU/kg) had an initial effect that was not sustained, suggesting stoichiometric excess of prothrombin vs dabigatran may be required
Honickel et al., 2015 [18]	Porcine polytrauma model	30 (daily oral dose) then intravenous infusion to reach supratherapeutic plasma concentration	30 or 60	Significant decreases in PT, CT and CFT	Three-factor and four-factor PCCs are similarly effective for dabigatran reversal
Honickel et al., 2015 [58]	Porcine polytrauma model	30 (daily oral dose) then intravenous infusion to reach supratherapeutic plasma concentration	25, 50 or 100	50 and 100 IU/kg PCC associated with significant reductions in blood loss vs placebo group and those treated with 25 IU/kg	PCC can be effective in reducing blood loss in anticoagulated pigs
					High doses may induce a procoagulant state
					Low doses may be ineffective
				High-dose PCC (100 IU/kg) led to overcorrection of thrombin generation	

*aPCC* activated prothrombin complex concentrate, *aPTT* activated partial prothrombin time, *CFT* clot formation time, *CT* clotting time, *PCC* prothrombin complex concentrate, *PT* prothrombin time

**Table 4:** Clinical studies investigating the use of PCCs and aPCCs to reverse dabigatran-induced anticoagulation

Reference	Study design	Dose		Main results	Conclusion
		Dabigatran (mg)	PCC (IU/kg)		
Eerenberg et al., 2011 [59]	Randomised, placebo-controlled crossover (study included rivaroxaban and dabigatran)	150	50 (administered to healthy volunteers)	50 IU/kg PCC did not correct aPTT, thrombin-generation lag time, TT or ECT	PCC did not neutralise the anticoagulant effect of dabigatran
Marlu et al., 2012 [60]	Ex vivo, randomised crossover (study included rivaroxaban and dabigatran)	150	PCC (in vitro): 12.5, 25 or 50aPCC (in vitro): 20, 40 or 80	PCC restored changes in ETP at all three doses	Some non-specific reversal agents appear able to reverse the anticoagulant activity of dabigatran
				aPCC corrected both ETP and lag time at doses of 40 and 80 U/kg but not 20 U/kg	
Herrmann et al., 2014 [48]	Ex vivo, cohort study of patients receiving dabigatran for non-valvular atrial fibrillation	150	PCC (in vitro): 500 mU/mlaPCC (in vitro): 500 mU/ml	Dabigatran prolonged aPTT, PT, TT, dynamic parameters of TEG^®^ and ROTEM^®^ and thrombin-generation lag time; it also reduced ETP and thrombin-generation peak height	Some non-specific reversal agents appear able to reverse the anticoagulant activity of dabigatran
				All parameters ameliorated by aPCC	
				All parameters except PT ameliorated by PCC	

*aPCC* activated prothrombin complex concentrate, *aPTT* activated partial prothrombin time, *ECT* ecarin clotting time, *ETP* endogenous thrombin potential, *PCC* prothrombin complex concentrate, *PT* prothrombin time, *TT* thrombin time

**Table 5:** Case studies investigating the use of PCCs and aPCCs to reverse dabigatran-induced anticoagulation

Study	Patient	Dabigatran dose	Case presentation	Treatment	Results
Dumkow et al., 2012 [23]	85-year-old male with hypertension	150 mg twice daily	Acute liver failure, acute kidney injury and anaemia, with upper GI bleeding from an ulcer	2000 U PCC	Haemoglobin concentration stabilised and bleeding ceased
				16 U FFP	
Weitz et al., 2012 [32]	78-year-old male with AF, hypertension and a history of ischaemic stroke	150 mg twice daily	Haematemesis and melena	8 U RBCs	Blood loss was promptly reduced and the patient was stabilised
			Hb 5.9 g/dl	12 U platelets	
			Creatinine clearance 26 ml/min	8 U cryoprecipitate	Patient discharged on reduced dabigatran dose (75 mg twice daily)
			aPTT 83 s	40 U/kg PCC	
			TT > 150 s		
Javedani et al., 2013 [46]	54-year-old male with AF and hypertension	150 mg twice daily	Acute ischaemic stroke	4520 mg PCC	Coagulation parameters measured post PCC administration:
			Creatinine 1.0 mg/dl	1 mg rFVIIa	
			aPTT 30.3 s		aPTT 28.5 s
			INR 1.25		INR 0.82
					Patient was discharged after 7 days on aspirin and warfarin
Schulman et al., 2014 [61]	84-year-old male with AF	110 mg (unknown frequency)	Subdural haematoma following a fall	50 U/kg aPCC	No immediate change in coagulation profile
			TT 127 s		Thrombin time normalised after 3 days
			aPTT 46 s		Bleeding resolved
			INR 1.2		Patient discharged on day 4 with complete resolution of weakness
	81-year-old female with AF and hypertension	110 mg (unknown frequency)	CT scan identified haemorrhage in left basal ganglia	42 U/kg aPCC	Repeat imaging after 3 days showed slight increase in haematoma size
			aPTT 48 s		Speech normalised on day of admission
			TT > 150 s		Motor function required 2 months rehabilitation
					Normal mobility and strength but slight right-sided numbness after 13 weeks
	85-year-old female with AF, hypertension, dyslipidaemia, chronic kidney disease and previous myocardial infarction	75 mg twice daily	Undergoing insertion of dual-chamber pacemaker	100 U/kg aPCC	Bleeding ceased but thrombin time remained immeasurable for 3 days
			aPTT 65 s		
			Creatinine clearance 27 ml/min		
	83-year-old female with AF	110 mg twice daily	Admitted to hospital with upper GI bleeding	50 U/kg aPCC	Clinical condition stabilised following administration of PCC
				3 U RBCs	
			Hb 99 g/l		
			Creatinine clearance 24 ml/min		
Masotti et al., 2015 [62]	93-year-old female with AF	110 mg twice daily	Major bleeding from GI tract	25 U/kg PCC at 0 and 6 h	Bleeding ceased, but no improvement in coagulation parameters was observed after either PCC dose
			aPTT 89 s		
			PT 21 %	Tranexamic acid	
					No more re-bleeding occurred
					Coagulation parameters normalised after 6 days (aPTT 28 s, PT 90 %) and patient was discharged

*AF* atrial fibrillation, *aPCC* activated prothrombin complex concentrate, *aPTT* activated partial prothrombin time, *FFP* fresh frozen plasma, *GI* gastrointestinal, *Hb* haemoglobin, *INR* international normalised ratio, *PCC* prothrombin complex concentrate, *PT* prothrombin time, *RBC* red blood cell, *rFVIIa* recombinant activated factor VII, *TT* thrombin time

### Pre-clinical animal studies

A mouse model of intracerebral haemorrhage was developed to test the efficacy of PCC in preventing haematoma growth associated with dabigatran treatment [54]. Compared with controls, dabigatran etexilate (9.0 mg/kg; *n* = 391) significantly increased the haematoma volume (17.0 mm^3^ vs 11.9 mm^3^; *p* = < 0.05). The administration of 100 U/kg PCC, 30 minutes after the induction of intracerebral haemorrhage, effectively prevented dabigatran from increasing the haematoma volume (11.7 mm^3^; *p* < 0.05 vs dabigatran alone) and significantly reduced 24-hour mortality (4 % vs 30 % in mice receiving dabigatran alone; *p* < 0.05) [54].

Another study assessed the efficacy of PCC in the treatment of dabigatran-induced anticoagulation in a rabbit model [55]. Rabbits were given 0.4 mg/kg dabigatran (*n* = 20) and, 5 minutes later, PCC (20, 35 or 50 IU/kg) or placebo. After another 5 minutes, a standardised kidney incision injury was created. Compared with placebo, PCC produced a dose-dependent reduction in blood loss and acceleration in haemostasis.

A more recent study in rabbits assessed the efficacy of PCC for the treatment of dabigatran-induced anticoagulation, using different doses of both drugs [56]. Dabigatran was administered to anaesthetised rabbits as a 0, 75, 200 or 450 μg/kg bolus dose, followed by continuous infusion to maintain the post-bolus plasma dabigatran level. After 15 minutes, animals were given PCC doses of 0, 50 or 300 IU/kg and subjected to standardised kidney injury. The increase in blood loss induced by dabigatran was effectively attenuated by PCC. In addition, the thromboembolic risk associated with PCC (measured by histopathological examination of lung, kidney, heart and brain tissue) was reduced by the presence of dabigatran [56].

Both PCC and aPCC have been shown to be effective in treating the anticoagulant effect of dabigatran ex vivo in a porcine trauma model [49]. Dabigatran etexilate was given to animals (*n* = 5) orally for 3 days prior to surgery and intravenously on day 4 to achieve a supratherapeutic plasma dabigatran concentration (mean 1423 ng/ml). Following standardised blunt liver injury, blood samples were taken and PCC or aPCC (concentrations equivalent to doses of 30 and 60 U/kg) was added ex vivo. Both PCC and aPCC diminished the effects of dabigatran and trauma-induced coagulopathy. No differences were observed between PCC and aPCC [49].

In a similar porcine model of trauma with dabigatran anticoagulation, some animals received tranexamic acid (20 mg/kg) and fibrinogen concentrate (80 mg/kg) following trauma, before ex-vivo administration of PCC [18]. PCC administration effectively reduced dabigatran-induced anticoagulation, as shown by significant decreases in PT, EXTEM CT and EXTEM CFT. PCC also yielded additional coagulation improvements among animals treated with tranexamic acid and fibrinogen concentrate. The ex-vivo study design precluded assessment of the effects of PCC/aPCC on blood loss; consequently, two additional studies were performed using the same model of injury, with in-vivo administration of PCC or aPCC.

In the first of these studies, animals receiving dabigatran and polytrauma were randomly assigned to receive saline control or aPCC (25 or 50 IU/kg) [57]. Blood loss was not significantly different between animals in the control and 25 IU/kg aPCC groups (3807 ml and 3690 ml, respectively); however, 50 IU/kg aPCC was associated with blood loss of 1639 ml, a statistically significant reduction vs control and 25 IU/kg aPCC (*p* < 0.0001) [57]. aPCC was shown to produce improvements in PT, in CT and CFT in the EXTEM and INTEM assays and in thrombin-generation peak height and ETP; but not in aPTT or ACT.

Three doses of PCC (25, 50 or 100 IU/kg (PCC25, PCC50 or PCC100)) were compared with saline control in the second study [58]. Total blood loss was significantly lower in animals receiving the higher doses of PCC (PCC50 and PCC100: 1749 ml and 1692 ml, respectively) than in the control and PCC25 groups (3855 ml and 3588 ml, respectively). All doses of PCC improved the coagulation parameters INTEM CT, EXTEM CT and EXTEM CFT vs controls. In addition, PCC50 and PCC100 produced sustained increases in ETP [58]. Overall, this study showed that PCC25 produced similar outcomes to control, with only PCC50 and PCC100 reducing blood loss, but that PCC100 is associated with a procoagulant state. The study also confirmed the low sensitivity of certain coagulation parameters (e.g. ACT, aPTT, thrombin-generation lag time) to the effects of PCC on dabigatran anticoagulation.

In summary, these pre-clinical studies have demonstrated that sufficient doses of both PCC and aPCC (50 IU/kg) can successfully reverse the effects of dabigatran-induced anticoagulation in the pre-clinical setting, as shown by improvements in coagulation parameters, blood loss and mortality. The lack of effectiveness of lower doses may be explained by insufficient increases in thrombin generation. These studies do not show any significant differences between PCC and aPCC in the reduction of bleeding or the degree of thrombin generation. In addition, the lack of sensitivity of the aPTT test emphasises the importance of selecting an appropriate coagulation test for monitoring dabigatran reversal by PCCs or aPCCs [18, 49]. It is important to note that in the porcine studies the examined doses of PCC and aPCC were sufficient to control bleeding under high dabigatran concentrations; lower doses would almost certainly be adequate with lower, therapeutic plasma concentrations of dabigatran.

### Clinical studies: healthy volunteers

The first study to assess PCCs for the treatment of dabigatran-induced anticoagulation in humans was a randomised, placebo-controlled crossover study [59]. Healthy male volunteers (*n* = 12) were given either 20 mg rivaroxaban or 150 mg dabigatran twice daily, followed by 50 IU/kg PCC or saline. Following a suitable washout period, study participants were switched to the other anticoagulant. The coagulation tests aPTT, ETP, lag time, TT and ECT were performed to measure the effects of PCC on the anticoagulation induced by dabigatran. Dabigatran prolonged the aPTT (*p* < 0.001), thrombin-generation lag time (*p* < 0.001), TT and ECT (*p* = 0.002) in all subjects. The 50 IU/kg dose of PCC was not sufficient to normalise any of these parameters in healthy volunteers [59]. This result is attributable to the use of endpoints that are not sensitive to PCC reversal of dabigatran [49]; also, there may be differences between healthy volunteers without coagulopathy and clinical patients in whom emergency dabigatran reversal is needed.

In a randomised, crossover, ex-vivo study, 10 healthy male subjects were randomised to receive one oral dose of either dabigatran (150 mg twice daily) or rivaroxaban (20 mg daily) [60]. After a 2-week washout period, each patient received the other anticoagulant. In dabigatran-treated patients, PCC added to blood samples in vitro effectively reversed dabigatran-induced changes in the ETP at all three doses (12.5, 25 and 50 IU/kg); aPCC was able to correct both the ETP and thrombin-generation lag time at doses of 40–80 U/kg [60].

### Clinical studies: patients

In 17 patients receiving 150 mg dabigatran twice daily for AF following hip or knee arthroplasty, plasma samples were taken to characterise the anticoagulant effect [48]. In addition, the effects of ex-vivo addition of PCCs and aPCCs were investigated using blood samples. Dabigatran administration significantly prolonged a number of laboratory parameters, including the PT, aPTT and TT. In addition, the TEG^®^ parameters of reaction time and alpha angle and the ROTEM^®^ parameters INTEM CFT and EXTEM CFT were all significantly increased. Assessment of thrombin generation using a CAT (Thrombinoscope BV, Maastricht, the Netherlands) showed that the lag time was increased and ETP was decreased. Ex-vivo addition of 500 mU/ml PCC significantly ameliorated all parameters with the exception of the PT, and addition of 500 mU/ml aPCC (FEIBA) ameliorated all parameters [48].

### Case reports

Several case reports assessing the efficacy of PCCs and aPCCs for treatment of bleeding associated with dabigatran-induced anticoagulation have been published. Case-report outcomes constitute low-quality data and cannot be considered conclusive because of the small numbers of patients and variations in aspects such as type of bleeding, doses of PCC/aPCC and co-morbidities of the patients. In addition, individual patients are not usually treated with PCC or aPCC alone, so clinical outcomes are not necessarily determined specifically by these agents. Despite these limitations, we consider the published case reports to be worthy of consideration.

In a case series of four patients with life-threatening dabigatran-related bleeding (subdural haematoma, intracerebral haemorrhage, surgery for insertion of a pacemaker and gastrointestinal bleeding), the administration of aPCC (dose range 42–100 U/kg) was associated with bleeding being brought under control [61]. A number of other case reports have also reported successful outcomes following treatment with PCC or aPCC as a response to ischaemic stroke (embolic thrombus of the left middle cerebral artery) [46] or gastrointestinal bleeding [23, 32, 62] occurring in patients under dabigatran treatment. However, in some of these cases rFVIIa was administered as concomitant therapy, making the specific impact of PCC/aPCC on dabigatran anticoagulation difficult to estimate. In contrast, there have also been cases where the use of PCC alongside rFVIIa has appeared ineffective, with no improvement of coagulation parameters or reduction of bleeding [63].

## Safety of PCCs and aPCCs

The procoagulant/prothrombotic risks of treatment with PCCs and aPCCs must be weighed against the benefits. It should be noted that neither PCCs nor aPCCs are currently licensed for the treatment of DOAC-induced anticoagulation; therefore, any such use is off-label. The majority of evidence relating to the safety of PCCs has been obtained from VKA reversal or non-anticoagulated patients with perioperative bleeding. These are very different situations from the emergency treatment of DOAC-induced anticoagulation, and more evidence is needed regarding the safety of PCCs in this setting.

Historically, PCCs/aPCCs have been associated with a risk of thrombotic complications when used for the treatment of haemophilia or VKA reversal [64]. Composition adjustments, such as the inclusion of coagulation inhibitors, reduced use of activated factors and improved balance of coagulation factor content, have been implemented with the aim of improving the safety of PCCs [65]. Relative levels of factor II (prothrombin) and the key inhibitor antithrombin have been identified as the major cause of thrombogenicity [66]. In an observational study in trauma patients, PCC was shown to increase the ETP for 3–4 days following treatment (i.e. approximately the half-life of prothrombin) [67]. In addition, patients receiving PCC had low levels of antithrombin. It has been suggested that PCCs should be labelled according to prothrombin content, rather than factor IX content [66]. Overall, the available safety data indicate that there are possible risks when using PCCs.

An in-vivo animal study assessed the safety of PCC for the treatment of dabigatran-induced anticoagulation [56]. In the absence of dabigatran, high-dose PCC (300 IU/kg) produced low-grade pulmonary emboli in 5/5 (100 %) of animals. However, when the same dose of PCC was administered to animals previously treated with dabigatran, the frequency of pulmonary emboli was decreased in the presence of dabigatran in a dose-dependent manner (2/5 (40 %) at a dabigatran dose of 75 μg/kg, 1/5 (20 %) at 200 μg/kg and 0/5 (0 %) at 450 μg/kg) [56].

In a porcine model of coagulopathy with blunt liver injury and no anticoagulation, administration of a four-factor PCC (50 IU/kg) resulted in protracted elevation of thrombin–antithrombin complexes and D-dimers, and formation of thromboemboli and pulmonary fibrinogen deposits in some animals [68]. Signs of disseminated intravascular coagulation were also shown in 44 % of animals. In contrast, a PCC dose of 35 IU/kg safely improved coagulation parameters and halted blood loss. Furthermore, survival and total blood loss were significantly improved in both PCC groups when compared with control animals [68].

In contrast to the previous study, the administration of PCCs or aPCCs to pigs after blunt liver injury under high-dose dabigatran appears not to be associated with thromboembolic events. Histopathological assessments showed that there was no thrombus formation in the heart, lungs, liver and kidneys after administration of PCC (25, 50 or 100 IU/kg) [58] or aPCC (25 or 50 IU/kg) [57].

## Clinical perspective and conclusion

DOACs such as dabigatran have proven effective in decreasing the risk of ischaemic stroke in patients with AF, and in the prevention and long-term treatment of VTE. Although the bleeding risk associated with dabigatran is low, any anticoagulant can cause bleeding. With life-threatening bleeding in dabigatran-treated patients, urgent reversal of the thrombin inhibitory effects of dabigatran should be considered. The available data indicate that PCCs and aPCCs may be able to reverse dabigatran-induced anticoagulation in a dose-dependent manner. However, we do not have high-level evidence to support the use of PCCs/aPCCs in this setting, so the recommendation to use them is based on haematological principles, animal studies, healthy volunteer studies, an ex-vivo study of ‘real-world’ patients, and outcomes in a few case reports.

Treatment with PCCs or aPCCs increases the concentrations of several coagulation factors, including prothrombin which has a half-life of 60–72 hours [51]. Thrombin generation may therefore be enhanced for several days after the use of PCCs to treat major bleeding [67]. This may be associated with an increased risk of thromboembolic events. The lack of high-level evidence with PCCs and aPCCs for dabigatran reversal makes it difficult to make dose recommendations, but it appears necessary to use the minimum effective dose. On the other hand, there is evidence that low doses may not be effective in reversing the anticoagulant effects of dabigatran, probably because of the need to increase the plasma concentration of thrombin to that of dabigatran [58]. This scenario is complicated by the lack of specific coagulation tests that are routinely available and rapid to perform, with established sensitivity and specificity for guiding the required dose of PCC or aPCC and then monitoring the effects of treatment. There is a clear need for a test that fulfils these criteria. Against this background, it is unsurprising that there are variations between guidelines for the use of PCCs/aPCCs in the management of bleeding among dabigatran-treated patients. Treatment decisions will be made on a case-by-case basis according to clinical judgement, local hospital protocols and product availability. Because of the theoretical thromboembolic risk, it is reasonable to titrate PCCs/aPCCs according to the clinical condition of the patient, starting with an initial dose of 25 IU/kg. However, clinical data are needed to establish the optimum dosing strategy. Because the mechanisms of action of PCCs and aPCCs are similar (both act by increasing thrombin generation), there is no need to use first one and then the other in a step-wise approach to bleeding management. It is essential to remember that the anticoagulant effect of dabigatran may be only one aspect contributing to coagulopathy; the likelihood of coexistent hyperfibrinolysis, dilutional coagulopathy and loss of coagulation factors, etc. [69] require a multi-therapeutic approach. In the future, once the specific reversal agent idarucizumab becomes widely available, this treatment will be considered preferable to PCCs and aPCCs for dabigatran reversal because it has not been associated with a risk of thromboembolic events and has shown no procoagulant effect in various laboratory analyses. The phase III RE-VERSE AD study of idarucizumab showed complete reversal of the anticoagulant effect of dabigatran within minutes, in patients with serious bleeding or who required an urgent procedure [19]. However, idarucizumab may not be available at every hospital for quite some time. Also, there could conceivably be clinical circumstances under which PCCs or aPCCs might be valuable as part of multimodal therapy, such as when thrombin generation is impaired as a result of trauma-induced coagulopathy [70], although this needs to be evaluated in clinical studies.

## Conclusions

Certainly at present, PCCs and aPCCs are far more widely available than idarucizumab, meaning that their use for emergency reversal of dabigatran among bleeding patients may be warranted. The available data do not provide evidence that either PCCs or aPCCs should be considered preferable to the other. RCTs are required to provide a robust evidence base, to ascertain the optimum dosing strategy and to determine the relative effectiveness of PCCs and aPCCs in this setting.

## Abbreviations

ACT: activated clotting time
AF: atrial fibrillation
aPCC: activated prothrombin complex concentrate
aPTT: activated partial thromboplastin time
CAT: calibrated automated thrombogram
CFT: clot formation time
CT: clotting time
DOAC: direct oral anticoagulant
dTT: diluted thrombin time
DVT: deep vein thrombosis
ECA: ecarin chromogenic assay
ECT: ecarin clotting time
ETP: endogenous thrombin potential
INR: international normalised ratio
PCC: prothrombin complex concentrate
PT: prothrombin time
RCT: randomised controlled trial
rFVIIa: recombinant activated factor VII
r-TEG: Rapid TEG
TT: thrombin time
VKA: vitamin K antagonist
VTE: venous thromboembolism
